# A Bibliometric Analysis of Immunological Research in Primary Biliary Cholangitis: Global Patterns and Emerging Directions

**DOI:** 10.7759/cureus.89942

**Published:** 2025-08-12

**Authors:** Wentao Zhang, Bing Xu, Danfeng Ren, Yingren Zhao, Jinfeng Liu

**Affiliations:** 1 Department of Infectious Diseases, The First Affiliated Hospital of Xi'an Jiaotong University, Xian, CHN

**Keywords:** bibliometric analysis, bile acids, immune cells, predictive factors, primary biliary cholangitis

## Abstract

Despite the significant advances in autoimmune research, primary biliary cholangitis (PBC) remains a clinically challenging and incompletely understood disease, with its exact causes and molecular mechanisms still elusive. The immune system exerts crucial pathogenic effects throughout PBC development, from initial biliary injury to end-stage cirrhosis. Despite decades of investigation, the current understanding of PBC pathogenesis remains fragmented, particularly regarding the immune mechanisms involved in the disease. To address this, we conducted a bibliometric analysis of immunological studies on PBC, utilizing data from the Web of Science database, which covers publications from 1970 to 2023, and identified 2,042 studies for analysis. The findings revealed a steady increase in research activity over the decades. Our analysis revealed a consistent upward trend in PBC-related studies, with an average growth rate of 14.45%. The United States leads as the top contributing country, with the University of California, Davis, and Dr. Gershwin M. Eric emerging as the most influential institution and author, respectively. Among journals, Hepatology is the most prominent, with the highest number of citations and co-citations. Recent advances in diagnostic techniques have substantially improved early detection rates of PBC while dramatically decreasing instances of disease decompensation. This paradigm shift has redirected research priorities toward innovative disease management strategies and patient-centered care approaches. This synthesis presents a unified framework for PBC immunopathogenesis, pinpointing critical knowledge gaps and high-potential research trajectories in disease mechanisms and therapeutic innovation.

## Introduction and background

Primary biliary cholangitis (PBC), formerly known as primary biliary cirrhosis, exemplifies a canonical autoimmune liver disease (a group of chronic immune-mediated hepatic disorders characterized by dysregulated self-tolerance) driven by multifactorial interactions between genetic susceptibility and environmental exposures. It disproportionately affects women, with a 4:1 female-to-male incidence ratio [1]. Growing recognition of PBC has led to rising reported prevalence worldwide, with high prevalence in North America and Northern Europe [2]. The disease is characterized by the progressive destruction of intrahepatic bile ducts, leading to cholestasis (impairment of bile formation and flow), progressive fibrosis, and ultimately end-stage biliary cirrhosis if untreated [3]. Ursodeoxycholic acid (UDCA) continues to be the internationally recommended first-line therapy for PBC [4]. At the same time, 40% of patients show inadequate biochemical responses, correlating with 50% lower 15-year transplant-free survival rates compared to responders [5]. For patients progressing to cirrhosis or developing refractory pruritus, liver transplantation constitutes the only definitive therapeutic option.

The increasing interest in this field is evident in the substantial number of research and review articles on it. Although some studies have provided preliminary insights into the trends and key topics in PBC [6,7], a more comprehensive bibliometric analysis is warranted to explore and understand the most active research domains and to discern shifts in research focus over time. In the present study, we aimed to systematically evaluate and map the research focuses related to the immunological mechanisms of PBC, and analyze the historical development of its key knowledge domains through an evaluation of the scientific literature. We additionally aimed to evaluate the PBC research ecosystem through network analysis of geographical, authorship, and publication patterns, while simultaneously identifying areas of research saturation, underexplored topics, and evolving scientific priorities.

## Review

Materials and methods

Search Strategies and Data Collection

**Figure 1:**
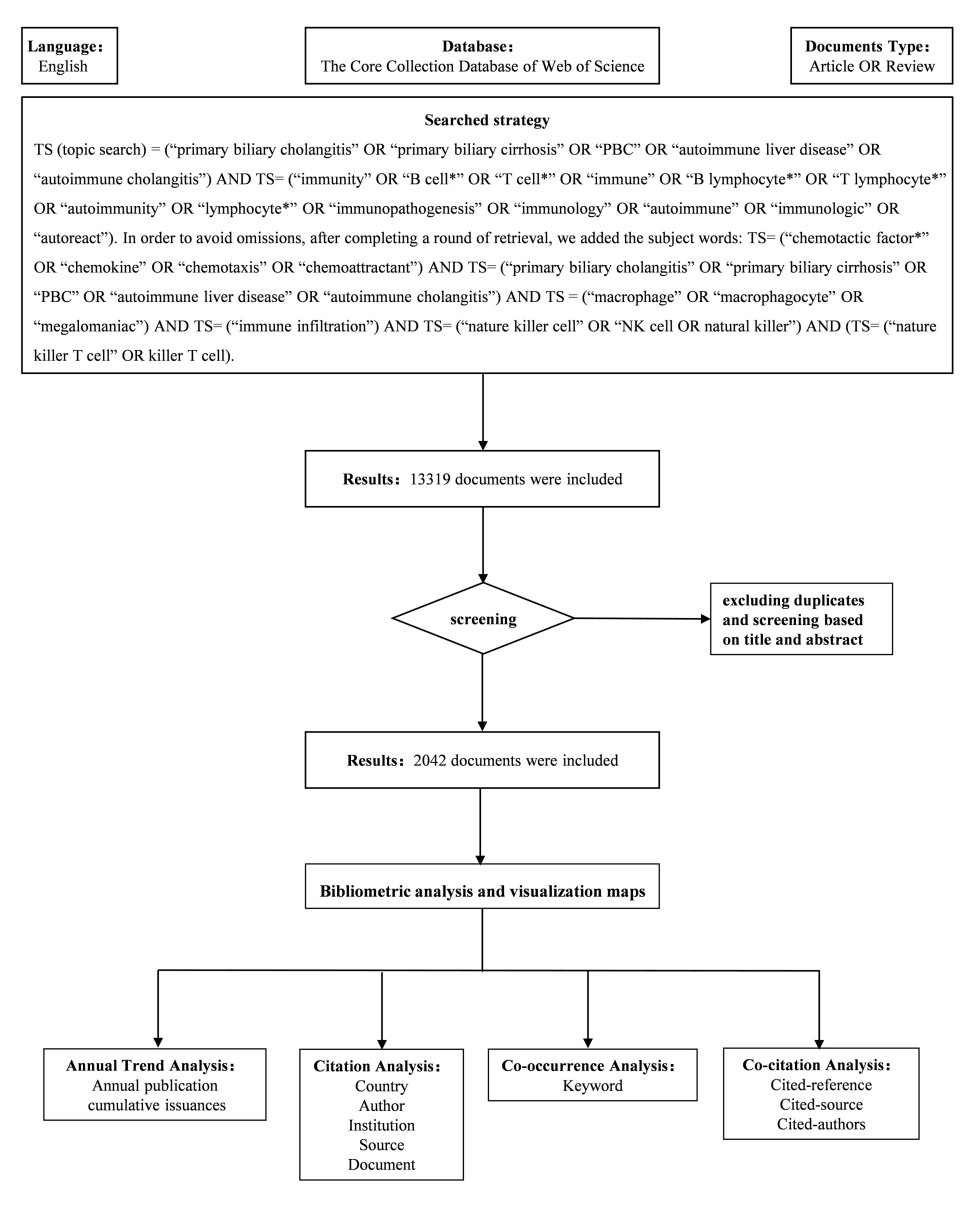
Literature screening and selection process Flowchart illustrating the systematic identification, screening, and inclusion of publications for bibliometric analysis, following PRISMA guidelines. A total of 13,319 records were initially retrieved from Web of Science (1970–2023). After duplicate removal and rigorous title/abstract screening, 2,042 publications (1,508 research articles; 534 reviews) were deemed eligible for analysis PRISMA: Preferred Reporting Items for Systematic Reviews and Meta-Analyses

The Web of Science Core Collection Database serves as an authoritative scientific database, encompassing both fundamental metadata (including authors, institutions, countries, and keyword terms) and unique citation network data (particularly cited references), which distinguishes it from other primary indexing services. Launched in 1970, the Science Citation Index (SCI) pioneered the standardized collection of citation data from indexed journals, establishing the first continuous bibliographic repository for scientific literature. To minimize citation count adjustments during retrieval, we restricted the Web of Science Core Collection search to research and review articles published between 1970 and November 30, 2023. Using Web of Science Boolean operators, we implemented subject filters that incorporated key terms, including primary biliary cholangitis, primary biliary cirrhosis, immunology, T cells, and B cells (see Figure 1 for the complete search strategy). Language was confined to English. The literature search, screening, and extraction were conducted independently by two authors (WTZ and DFR), with any disagreements resolved by JFL and YRZ. As shown in Figure 1, this resulted in 13,319 publications. After removing duplicates and applying stringent screening criteria, 2,042 publications were qualified for analysis, including 1,508 research articles and 534 review articles. Key bibliographic elements, including title, all authors, countries/regions, affiliations, references, number of citations, journals, publication date, and keywords, were automatically extracted for subsequent analysis.

Bibliometric Analysis and Visualization Maps

During data processing, author signatures were disambiguated through ORCID linkage, while keyword variants (e.g., 'primary biliary cirrhosis' and 'PBC') were standardized to 'primary biliary cholangitis' based on Medical Subject Headings terminology. Subsequently, the following analytical approaches were employed to identify research themes: (1) citation analysis of authors, countries, journals, publications, and institutions; (2) co-occurrence analysis of subject terms to map conceptual clusters [8]; and (3) to reveal foundational knowledge structures [9]. We implemented our analytical workflows using Microsoft Excel 2019 for data organization, R software (Version 4.4.3) for statistical computations, and VOSviewer (Version 1.6.19) for network visualization (including LinLog clustering and modularity optimization). Pajek (Version 5.18) and Scimago Graphica (Version 1.0.42) were executed to refine network visualizations.

Results

Global Publication Trends and Article Citation

**Figure 2:**
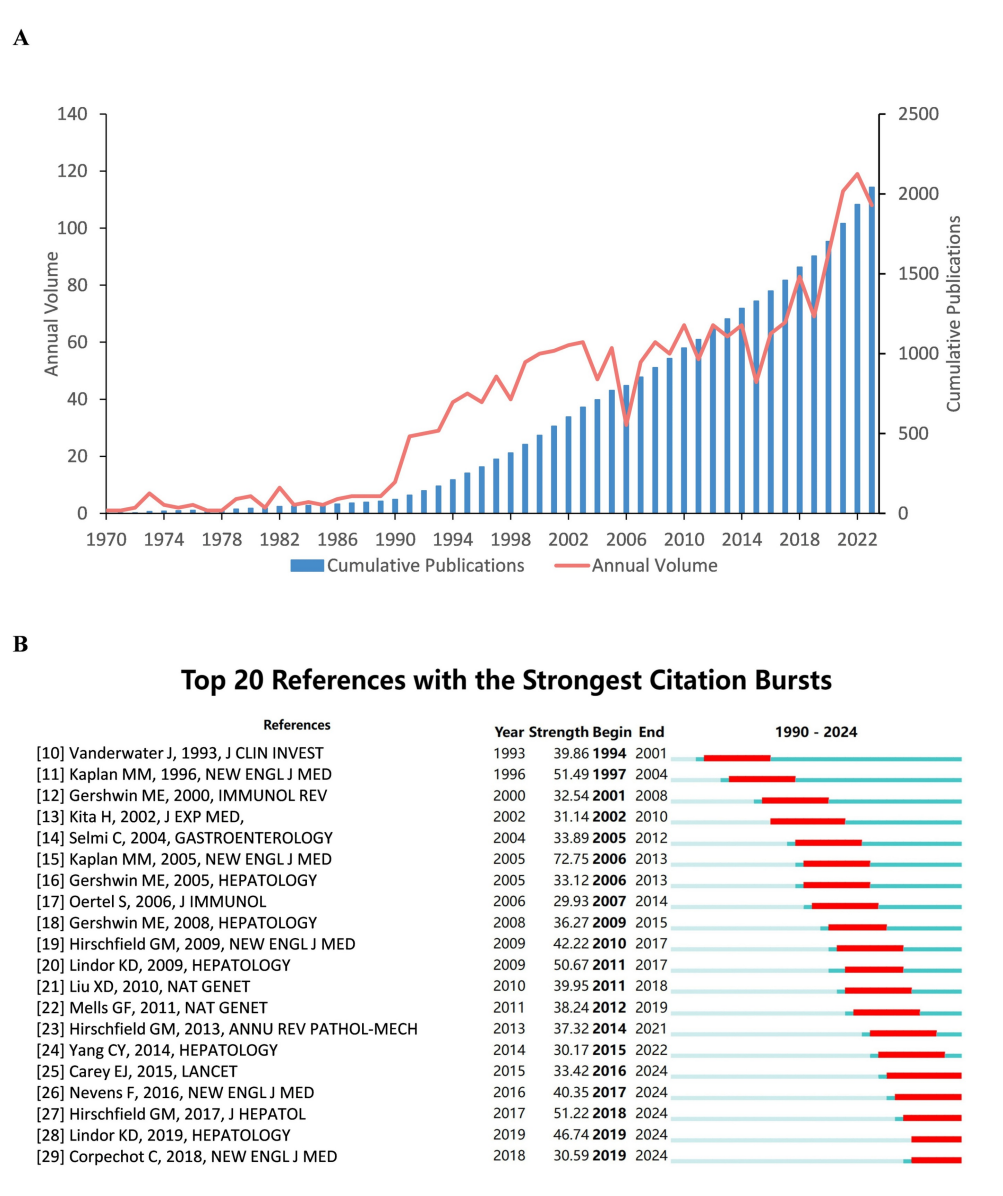
Annual Tendency and Citation Ranking* *[10-29] A. The annual number of publications and the cumulative number of publications. B. Top 20 references with the strongest citation bursts. In Figure B, the red segments represent the burst periods of citation growth

Using the specified search criteria, 2042 publications were retrieved spanning from April 1970 to November 2023. These publications involved 7,569 authors from 1,782 institutions across 61 countries, published in 437 journals, and garnered 60,727 citations from 4,947 journals. Figure 2A illustrates the global publishing trend over time. In terms of annual publication volume and cumulative annual publications, the publication rate has shown a consistent upward trajectory, albeit with fluctuations. The timeline reflects three evolutionary phases in PBC research according to the annual number of publications: (1) The early phase (1970 to 1990), marked by limited output and slow growth, reflecting the nascent stage of PBC research in immunology. (2) The transitional phase (1990 to 2015) exhibited considerable annual fluctuations, with growth following a jagged trajectory. Despite the emergence of Genome-Wide Association Studies, which enhanced genetic insights, research efforts remained fragmented. (3) The expansion phage (2015 to 2023) saw a dramatic surge in publications, with annual counts exceeding 100 after 2021, signaling accelerated research activity. This phase synthesized broader perspectives with sustained mechanistic investigations, fostering cross-disciplinary research that advanced a more holistic understanding of PBC.

From the 2,042 analyzed articles, 185 (9.1%) achieved over 100 citations, while all top 20 most-cited works exceeded 250 citations each (Figure 2B). Citation metrics identified Kaplan & Gershwin's 2005 seminal review 'Medical Progress: Primary Biliary Cirrhosis' as the most influential publication [15], with 7275 total citations - nearly 41.29% more than the second-ranked work. While our citation analysis identifies influential works, it is worth noting that some potentially transformative publications - particularly those published after 2020 - may be underrepresented due to insufficient time for citation accrual. 

Distribution and Connection of Countries/Regions and Institutions

**Figure 3:**
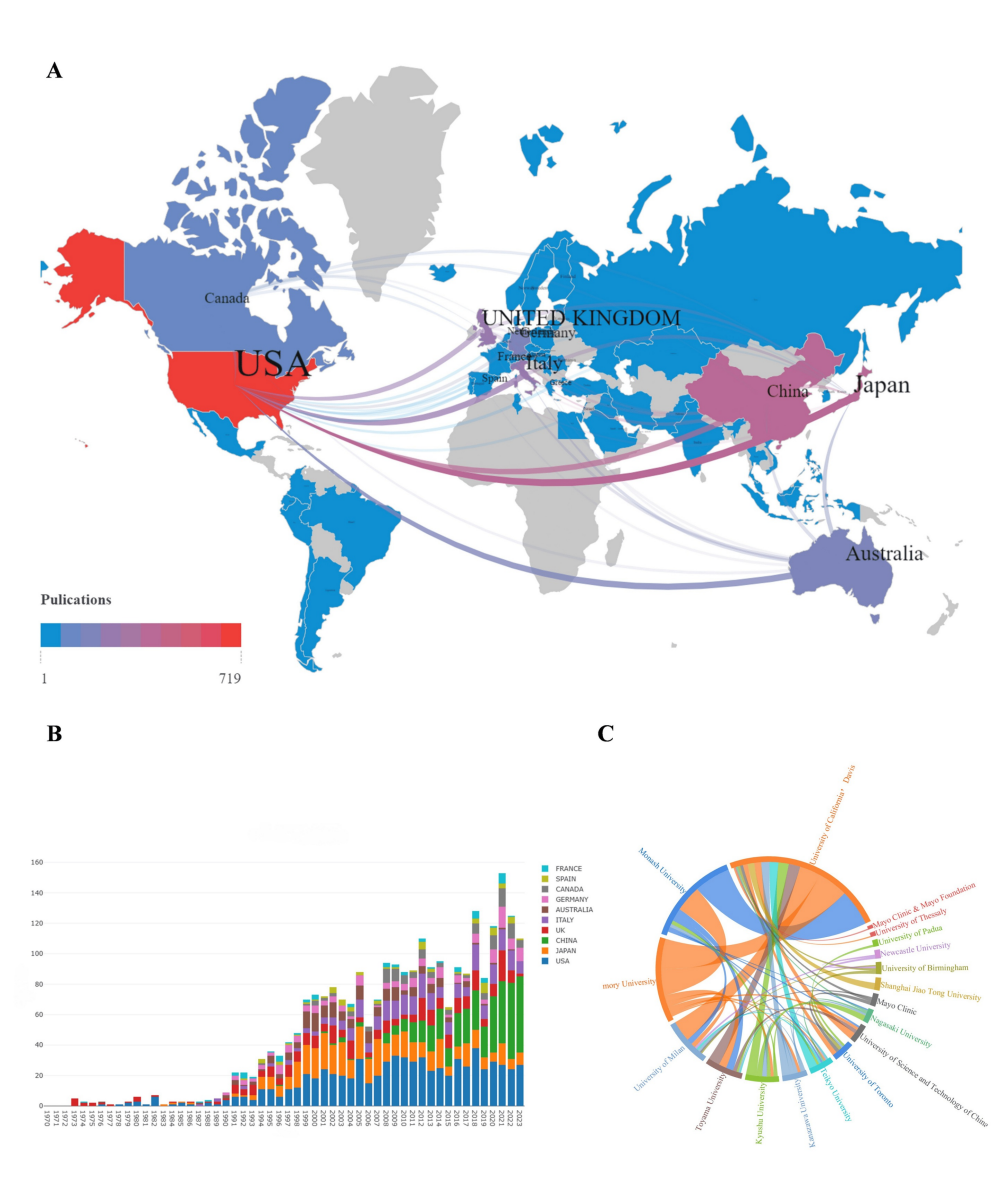
Visualization of national and institutional cooperation A. Geographical visualization of national publishing and cooperation. B. Changes in the number of papers published by the leading countries over the years. C. The string diagram of the cooperative relationship of institutions with more than 30 publications A: Geographic distribution of publication volume and collaboration strength. Color intensity represents the total publication output for each country. Line thickness indicates the strength of collaborative linkages between countries. B: Annual publication output composition by country. Stacked bar colors represent the proportional contribution of individual countries to the total yearly publication count. C: Institutional collaboration network among leading research organizations. The chord diagram illustrates cooperative relationships; colors denote distinct institutions, while connecting ribbons represent collaborative ties between them

Our geographic analysis revealed contributions from authors affiliated with institutions across 61 countries/regions, demonstrating the global scope of PBC research (Figure 3A). In the co-authorship network, connection thickness corresponds to the strength of collaboration between countries, while color intensity reflects the volume of publications.

The top 10 countries by publication output are listed in Table 1. Among them, the United States occupies a dominant position in research output, accounting for 36.26% of the total publications. Network analysis further revealed its pivotal roles as the primary hub for international collaborations, maintaining strong co-authorship ties with all other top-producing nations. The United States' dominance reflects intersecting advantages: (1) world-leading NIH funding for hepatology research, (2) a PBC prevalence rate of 40.2/100,000 - nearly triple the global median (14.6/100,000), and (3) concentration of 8 of the world's top 20 hepatology research centers. Temporal analysis revealed distinct national trajectories: The United States maintained a consistent output. Japan experienced peak publication activity from the 1990s to 2010, followed by a slight decline in recent years. In contrast, China’s publications began to surge significantly after 2008, exhibiting a progressive growth trend. Notably, China achieved research leadership after 2020, producing 35 annual PBC publications compared to 32 from the U.S. This transition coincided with a nearly 300% increase in hepatology funding from the National Natural Science Foundation of China and the establishment of more than 10 nationally accredited hepatology research hubs (Figure 3B).

**Table 1:** The top 10 countries by publication output TC: total citation

Country	Publications	TC
United States	734	37706
Japan	419	15401
China	379	8315
The United Kingdom	268	12217
Italy	249	10255
Australia	160	12160
Germany	151	5549
Canada	108	5700
Spain	63	3113
France	55	3927

Among the 1,782 analyzed institutions, only 54 institutions demonstrated high research output (more than 15 publications each), indicating concentrated productivity among a small subset of research organizations. Figure 3C mapped the collaborative relationships among institutions that produced more than 30 publications each, with node size reflecting publication volume and edge thickness indicating the frequency of collaboration. The top 10 institutions were geographically distributed across six nations: three each based in the United States and Japan, with single representatives from Canada, Australia, the United Kingdom, and Italy. Productivity analysis revealed clear leadership by the University of California, Davis (366 publications) in the United States, followed distantly by Monash University (140) in Australia and Emory University (99) in the United States. The citation impact was quantified using the average number of citations per publication (total citations divided by the publication count), with full results detailed in Table 2.

**Table 2:** Top 10 institutions with the most significant number of publications TC: total citation

Institutions	Publications	TC	TC/Publications
University of California, Davis	366	21325	58.27
Monash University	140	9876	70.54
Emory University	99	7907	79.87
University of Milan	93	5701	61.30
Kanazawa University	77	3571	46.38
University of Birmingham	65	3301	50.78
Kyushu University	61	3072	50.36
Teikyo University	56	1814	32.39
Mayo Clinic	55	4305	78.27
University of Toronto	50	3824	76.48

Citation analysis revealed Emory University as the impact leader (79.87 citations/publication), closely followed by Mayo Clinic (78.27) and the University of Toronto (76.48), indicating these institutions' disproportionate influence relative to publication volume.

Distribution and Connection of Authors and Sources

**Figure 4:**
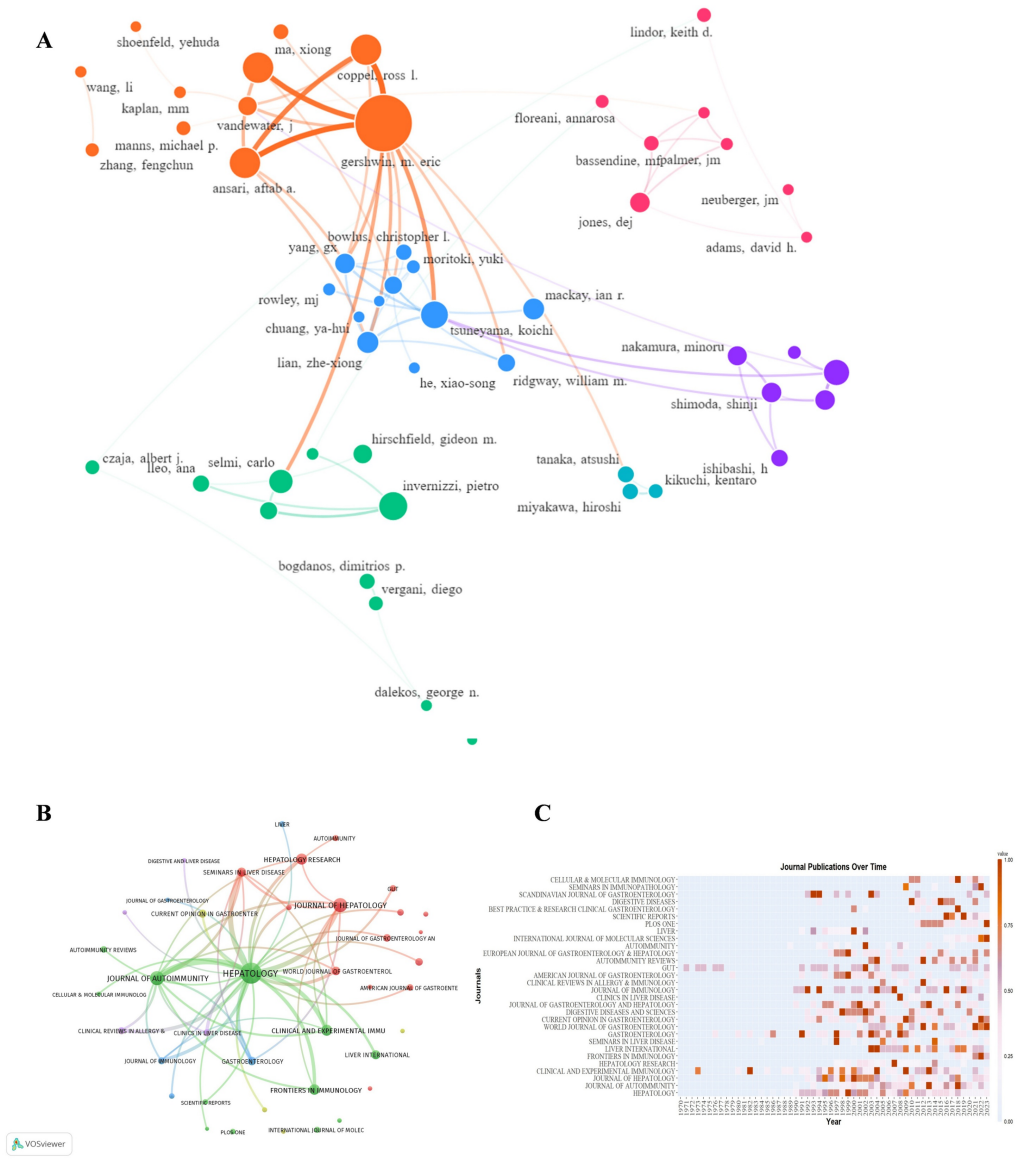
Visual analysis of authors and journals A. Author collaboration network. B. Cooperative relationship between journals. C. Number of manuscripts published in the top 30 journals over the years A: Node size represents individual author publication output (large central nodes indicate key scholars). Node color denotes automatic clustering by software. Edge thickness indicates collaboration strength between authors. B: Node size corresponds to journal publication volume. Edge color and thickness represent disciplinary relatedness and citation density between journals, respectively. C: Color gradient corresponds to publication density (darker hues indicate journals with higher publication output

Our analysis identified 7,569 unique authors in the field, with 50 demonstrating high productivity (i.e., more than 15 manuscripts each) (Figure 4A). This heavy-tailed distribution suggests a concentration of expertise among a small cohort of prolific researchers. Professor Gershwin ME (University of California, Davis) emerged as the most prolific contributor, with 343 publications-11.8 times the output of the median top-50 author (29 papers). The top 10 authors were geographically distributed across five nations: three from the United States, two each from Japan and Italy, and one from Australia and China, reflecting the field's global leadership structure.

The concentration of elite authorship mirrored institutional productivity: three top American authors hailed from the University of California, Davis (national publication leader). At the same time, both Australian representatives were affiliated with Monash University, consistent with these institutions' dominance in country-level output. These elite authors collectively drive the field’s intellectual trajectory, serving as corresponding authors on 68% of clinical guidelines (2010-2023). Multilevel collaboration across national borders, institutions, and research teams constitutes a fundamental prerequisite for conducting high-impact studies with adequate statistical power in PBC research.

The 2,042 publications appeared in 437 distinct journals, with 78% concentrated in gastroenterology (48%), hepatology (22%), and immunology (8%) journals, delineating the field's disciplinary boundaries. Among the 437 journals, only 65 (14.9%) published more than five manuscripts, indicating concentrated output within a core group of specialist periodicals (Figure 4B). Among the top 10 journals ranked by publication volume (Table 3), three were based in the United States and two in the United Kingdom. Hepatology (IF2022 = 14) ranked first with 184 publications, followed by the United Kingdom's Journal of Autoimmunity (IF2022 = 12.8) and the Netherlands' Journal of Hepatology (IF2022 = 25.7), each publishing more than 80 manuscripts. Figure 4C illustrates the temporal publication patterns among the top 30 journals, revealing their evolving contribution to the field's knowledge base.

**Table 3:** The number of citations and co-citations in the top 10 journals IF: Impact Factor

Journals	Publications	Citations	Co-citations	IF (2022)
Hepatology	180	15153	15549	14
Journal of Autoimmunity	88	4314	3230	12.8
Journal of Hepatology	87	4696	5908	25.7
Clinical and Experimental Immunology	59	2243	1902	4.6
Hepatology Research	55	733	532	4.2
Frontiers in Immunology	51	992	839	7.3
Liver International	41	1018	1042	6.7
Seminars in Liver Disease	41	1567	1273	4.2
Gastroenterology	37	3038	5778	29.4
World Journal of Gastroenterology	37	917	2038	4.3

Co-citation Analysis and Visualization

Co-citation analysis is a crucial bibliometric method that reveals collaborative patterns, research trends, and the knowledge structure in academic fields through examining document co-citation relationships. For instance, when two articles are consistently cited together, this often signifies their substantial impact within a particular research domain or their role in providing seminal theoretical works or crucial empirical references to address specific research challenges. In practice, co-citation analysis empowers researchers to identify foundational literature within a research domain, uncover potential collaborators through academic networks, and trace the evolution of thematic and theoretical frameworks. Moreover, it is a valuable tool for evaluating research impact, since highly co-cited literature generally indicates widespread academic acceptance and significant influence within the academic community.

**Figure 5:**
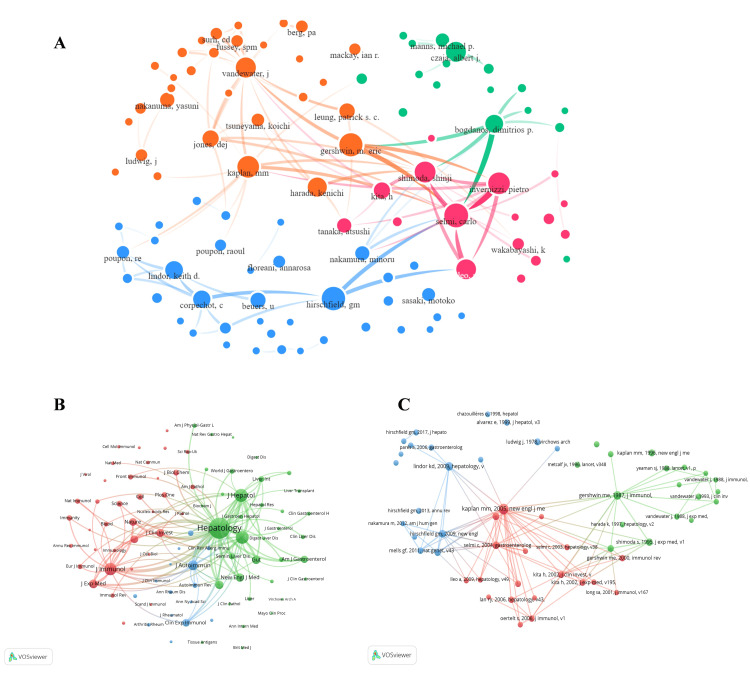
Co-author, co-journal, and co-reference visual network A. Co-author network with a total of more than 100 citations. B. Co-journal network with more than 200 citations. C. Co-reference network with a total of more than 80 citations Network analysis of co-citation patterns. Node size represents co-citation frequency. Node color denotes automatically generated clusters by the analysis software. Edge thickness indicates the association strength between nodes

The analysis identified 39,558 citations, of which 101 were cited more than 100 times (Figure 5A). Among the highly cited authors, Selmi Carlo, Gershwin M. Eric, and Hirschfield GM emerged as the most influential, each receiving over 800 citations. Regarding the co-citation of references, 60,723 cited articles were identified, including 140 highly influential works cited more than 50 times (Figure 5B). These co-cited articles were published across 4,945 distinct journals, of which 88 journals appeared more than 200 times each in the citation network (Figure 5C). The journal Hepatology ranked as the dominant journal, leading all publications with 15,153 citations and 15,549 co-citations in the analysis. The network visualization of journal co-citation relationships positioned Hepatology as the central hub, exhibiting the most robust bibliographic linkages to other prominent journals and the highest betweenness centrality in the field.

Co-occurrence Analysis of Keywords

**Figure 6:**
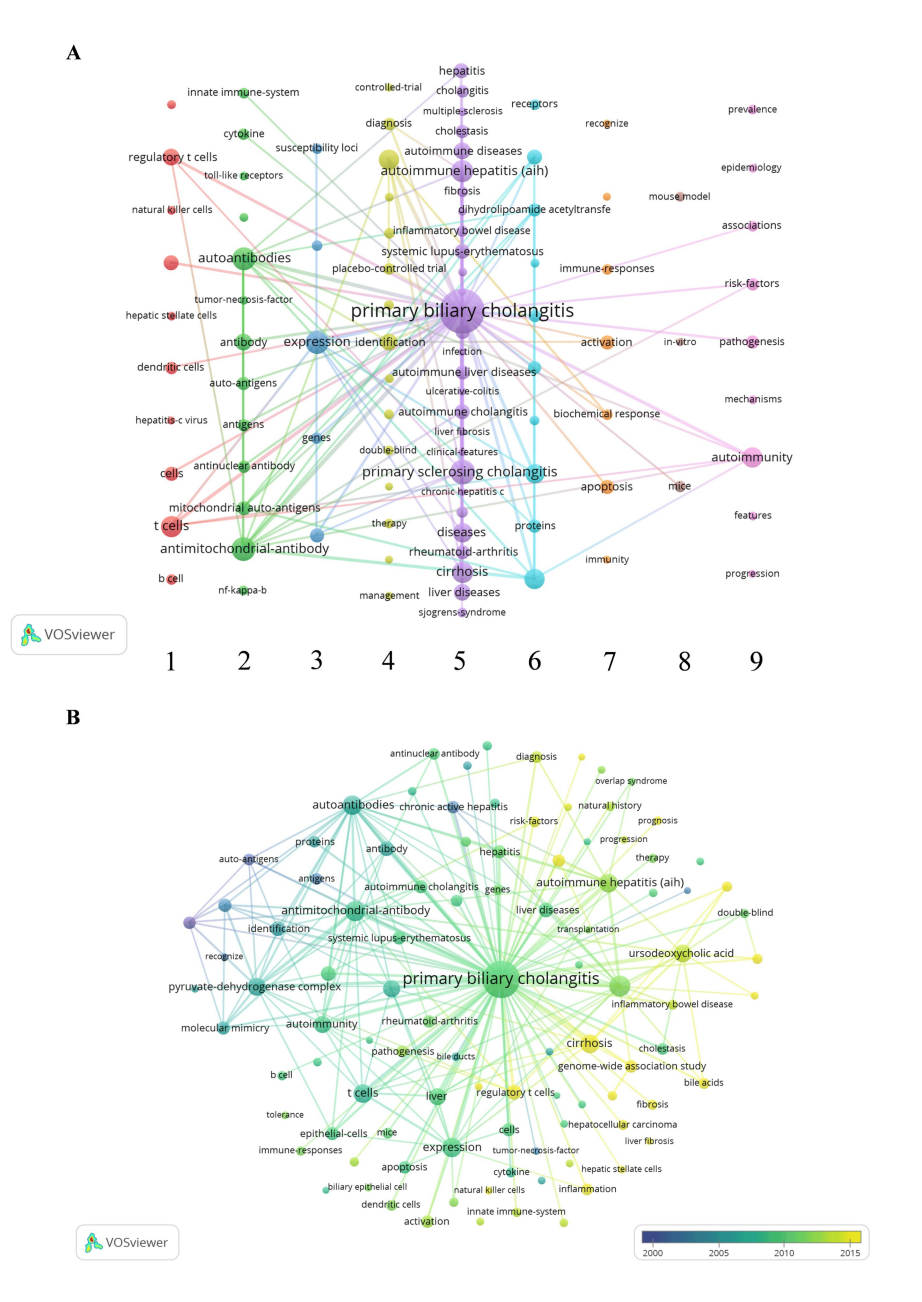
Keyword distribution and co-occurrence analysis A. Top 100 keywords co-occurrence visualization. B. Top 100 keywords time course evolution A. Thematic keyword clustering: Node color denotes thematic cluster membership of keywords. Node size represents keyword frequency. Edge thickness indicates co-occurrence frequency between keywords. B. Temporal keyword dynamics: Node color represents temporal activity of keywords (color gradient indicates emergence time). Node size corresponds to keyword influence. Edge color signifies persistence of associations (lighter hues: transient links; darker hues: stable long-term connections)

A total of 3710 words were extracted after merging synonym keywords, and the top 100 high-frequency keywords appeared more than 36 times each, indicating core research themes in the dataset (Figure 6A). These keywords were systematically clustered into nine distinct thematic topics: (1) Organisms (e.g., cells, viruses, bacteria); (2) Immune-related substances (e.g., antibodies, antigens, chemokines); (3) Genetics (e.g., genes); (4) Clinical relevance (e.g., drugs, diagnosis, treatment); (5) Macro-diseases (e.g., PBC, PSC); (6) Atypical immune or other substances (e.g., pyruvate dehydrogenase complex); (7) Cell behavior (e.g., apoptosis); (8) Experimental models (e.g., mice); (9) Non-specific terms (e.g., immunity, epidemiology). The size of each node scaled proportionally to the frequency of the keyword, while the strength of the associations between keywords determined the thickness of the edges. This keyword analysis reveals that frequency and co-occurrence patterns effectively map the intellectual structure of the field, identifying dominant research themes and emerging areas of focus, which also facilitates systematic detection of evolving research trajectories, revealing both established intellectual patterns and nascent thematic developments within the discipline.

The keyword network revealed distinct thematic clusters strongly associated with PBC. Cluster 1, dominated by T cells, Treg (regulatory T cells), cholangiocytes, and DC cells (dendritic cells), highlights the central role of immune dysregulation. Cluster 2, featuring AMA, autoantibodies, and mitochondrial autoantigens, underscores diagnostic and mechanistic markers. Cluster 3, anchored by expression, suggests gene- and protein-level investigations. Cluster 4, centered on UDCA, reflects its clinical prominence. Cluster 5 research on comorbidities related to PBC. Cluster 6, including the pyruvate-dehydrogenase complex, links mitochondrial dysfunction to PBC pathogenesis.

Given our emphasis on PBC immunology, Clusters 1 and 2 emerged as the most prominent thematic groups. Their large node sizes and dense interconnections reflect their dual role as (1) mechanistic drivers of immune-mediated bile duct injury and (2) diagnostic cornerstones in clinical practice. Temporal keyword analysis revealed distinct evolutionary patterns: autoantigens emerged as early research foci, while mechanistic terms, such as regulatory T cells and bile acids, gained prominence more recently (Figure 6B). This chronological shift mirrors the field's progression from diagnostic characterization to therapeutic and prognostic investigations, revealing PBC's now-established diagnostic criteria.

Considering keywords such as bile acids, genome-wide association studies, and regulatory T cells, which have gained increasing attention in recent years, this triad represents the evolution of PBC research toward mechanism-targeted interventions. While our high-frequency keyword analysis captures established research fronts, we acknowledge that nascent topics from the past two years may not yet meet this high-frequency threshold. We systematically address these emerging trends through the manual curation of recent literature, as detailed in the Discussion section.

Discussion

General Information

In this bibliometric investigation, we conduct a comprehensive analysis of the trajectory of PBC research spanning 5 decades. Our systematic analysis summarizes 2,042 PBC publications over the last 50 years. The geographical distribution reveals concentrated productivity from scientifically advanced regions, including the United States, East Asia, and Western Europe. This reflects both the technological capacity and the distribution of disease burdens across high-income countries. While China's annual PBC publications increased rapidly (2010-2020), the average citation impact (19.26 citations/paper) remains below that of US outputs (49.08 citations/paper), suggesting differences in research influence despite growing productivity.

From a historical perspective, the formal reclassification from 'primary biliary cirrhosis' to 'primary biliary cholangitis' (PBC) in 2015 marked a pivotal conceptual shift, replacing the misleading emphasis on cirrhosis with an accurate characterization of cholangiopathy, reflecting decades of pathological insights. Our analysis reveals a 38.91% increase in annual PBC publications (2015-2023 vs. 2006-2014), coinciding with the nomenclature shift, which suggests that the terminological clarity stimulated new investigative avenues. The 2017 monograph by Jansen et al.(cited 200+ times) fundamentally reframed PBC research priorities by introducing the 'ascending pathophysiology' model (bile acid toxicity → immune dysregulation) [30]. Systematic improvements in PBC diagnostics (with sensitivity now at 95%) and first-line therapies (UDCA response rates: 58.8-90%) have fundamentally altered the clinical landscape of PBC. A recent bibliometric analysis reveals a 210% increase (2015-2023) in studies addressing dynamic risk prediction, symptom stratification, and control of co-occurring symptoms. This paradigm shift highlights an evolution in clinical cognition, marking the transition of PBC research toward precision medicine and personalized therapeutic strategies.

Global Research Landscape Evolution

The United States maintains global dominance in PBC research, accounting for 36.26% (734/2,042) of international publications. Institutional leadership (e.g., University of California, Davis), individual leadership (e.g., Eric M. Gershwin), and journal-level leadership (e.g., Hepatology) demonstrate sustained research dominance through established academic networks. U.S. research comprehensively addresses PBC investigation, spanning from fundamental mechanisms (e.g., antimitochondrial antibodies [31]) to clinical applications (e.g., UDCA validation [32]), while serving as the hub of an international collaborative network with European and East Asian institutions (Figure 3A). While U.S. studies have significantly advanced PBC research, their predominant focus on Caucasian populations has resulted in limited representation of Asian genetic susceptibilities, potentially constraining the global generalizability of findings [19].

China now emerges as the third-largest producer of PBC research (379 publications, 18.73% of global output). This expansion coincides with a 58% increase in funding support (e.g., National Natural Science Foundation) and extensive clinical resources. However, Chinese institutional influence remains limited (e.g., University of Science and Technology of China vs. Emory University: 37.36 vs. 79.87 TC/Publications), limited by insufficient global research integration and methodological homogeneity (predominantly epidemiological investigations).

Japan made seminal contributions to PBC research during 1990-2010, most notably through: (1) development of the IL-2R alpha /CD25-deficient mouse model that revolutionized autoimmune cholangitis studies, and (2) comprehensive characterization of anti-gp210 autoantibodies as specific diagnostic markers [33,34]. Despite these foundational contributions, Japan's PBC research output has exhibited a paradoxical decline in recent years, likely attributable to shifting national research priorities toward aging-related disorders and the outflow of young investigators to other fields.

Temporal Evolution of Research Hotspots

Bibliometric analysis of keyword co-occurrence networks and citation trajectories reveals three transformative paradigm shifts in PBC immunology over the past five decades. The period spanning 1970-1990 witnessed a predominant research focus on diagnostic biomarker development and nosological characterization, with antimitochondrial antibodies (AMA) and the disease nomenclature 'primary biliary cirrhosis' establishing themselves as the field's defining conceptual anchors, epitomized by Mackay's seminal contributions (1987) [35], which established AMA-M2 antigens as the gold-standard diagnostic biomarkers for PBC, now incorporated into all major clinical guidelines. The subsequent phase (1990-2015) marked a critical shift toward immune mechanisms and genetic susceptibility, driven by advances in genomic technologies. Keywords such as Treg, Genome-Wide Association Study, and HLA-DR rose to prominence, reflecting a move from descriptive immunology to mechanistic exploration [21,22,36]. Notably, Hirschfield et al. identified the IL-12/STAT4 pathway as a pivotal driver of immune dysregulation, highlighting the role of Th17/Treg imbalance in disease pathogenesis [19,24,37].

Encouragingly, Treg cells demonstrate promising therapeutic potential: Tewari et al. engineered antigen-specific Tregs that effectively suppressed PDC-E2-reactive polyclonal CD4+ T cells from PBC patients in vitro [38,39]. Furthermore, clinical studies utilizing Good Manufacturing Practice (GMP)-grade Treg therapy for autoimmune liver diseases have demonstrated its safety profile [38,40]. However, determining the optimal dosing and timing for polyclonal GMP Treg infusion remains a significant challenge, indicating that translating the pathophysiological role of Tregs into routine clinical application still requires considerable exploration. The utilization of T cell receptor sequencing and chimeric antigen receptor (CAR) technology represents the next frontier for developing disease-specific CAR-Treg therapies.

Since 2015, research has entered an era of multi-omics integration and therapeutic innovation, with keywords like gut microbiome, bile acid metabolism, and FXR (farnesoid X receptor) signaling dominating the literature [41-43]. Jansen et al. identified bile acid toxicity as a central disease mechanism, further emphasizing its role in disease pathogenesis [30]. Obeticholic acid (OCA), a potent FXR agonist, was investigated as monotherapy in an international, randomized, double-blind, placebo-controlled phase 2 study. Based on promising interim data from this trial and others [44,45], OCA received FDA approval in 2016 as the first second-line therapy for PBC patients with an inadequate response to UDCA-nearly two decades after UDCA's initial approval. Subsequent evaluation of efficacy, safety, and durability in an ongoing open-label extension encompassed up to 6 years of treatment, yielding encouraging long-term results.

While recent studies have increasingly focused on combinatorial therapeutic strategies - such as OCA-fibrate combinations - and microbiome-targeted interventions, emerging evidence suggests the translation of mechanistic insights into clinically effective, disease-modifying treatments remains a critical challenge [46,47]. This persistent translational gap underscores an urgent imperative for well-designed longitudinal studies that systematically integrate genetic predisposition, metabolic dysregulation, and environmental triggers. This temporal trajectory-evolving from discrete biomarker identification to comprehensive systems biology approaches-encapsulates both the remarkable progress and persistent challenges in PBC immunopathology.

Research Gaps and Future Directions

Despite five decades of significant progress in PBC immunology, bibliometric analysis identifies persistent systemic limitations. Critically, the field remains fragmented by a key gap: the absence of an integrated framework elucidating crosstalk between immune cells (e.g., T cells, macrophages) and non-immune cellular compartments (e.g., cholangiocytes). Hisamoto et al. reported reduced anion exchanger-2 (AE2) expression on bile epithelial cells (BECs) in PBC patients requiring liver transplantation, which enhances migration of autologous splenic mononuclear cells toward BECs [48]. However, the generalizability of this finding is constrained-liver transplantation applies exclusively to end-stage disease (only 3 transplant recipients in our cohort), and contemporary pharmacotherapies have substantially reduced transplantation necessity. Notably, recent single-cell immunomics revealed a pivotal dichotomy: monocytes exhibit pro-inflammatory phenotypes in UDCA non-responders but adopt anti-inflammatory states in responders [49]. This functional divergence strongly implicates immune-non-immune cellular crosstalk as the core determinant of therapeutic response.

Current PBC immunological research is limited by a ‘single-cell paradigm’: although T-cell subset characterization (e.g., Th17/Treg) is well-established, the critical driver of bile duct-specific injury-immune cell-cholangiocyte crosstalk-remains a methodological blind spot. This gap stems from three barriers:(1) Technical: Scarcity of cholangiocytes, diminishing utility of liver biopsies in PBC management, inability of in vitro models to reconstruct 3D tubular architecture, and animal models lacking organ-specific pathology or recapitulating female predominance (90% of PBC patients are female) [50]; (2) Cognitive: Persistent focus on single-cell subset profiling while neglecting dynamic cellular dialogues; (3) Disease-related: Heterogeneity from factors like UDCA treatment history obscuring crosstalk signals.

This crosstalk specificity precisely underpins PBC’s bile duct tropism: cholangiocytes are not passive targets but actively participate in immune activation through antigen presentation (MHC-II), co-stimulatory signaling, and cytokine feedback. Confirming this, a 2023 Nature Communications study demonstrated significant ligand-receptor interactions between DUOX2⁺ACE2⁺ small cholangiocytes and immune cells (including B cells and plasma cells) in PBC patients [51], definitively establishing the pivotal role of immune-non-immune crosstalk in disease pathogenesis. With advancing spatial omics and organ-on-a-chip technologies, decoding the immune cell-cholangiocyte network offers a critical path to overcome therapeutic bottlenecks-such as identifying crosstalk-specific targets (e.g., polymeric immunoglobulin receptor) or developing localized immunological synapse inhibitors to avoid systemic immunosuppression.

Secondly, progress in developing early predictive biomarkers remains inadequate. Notably, research on non-invasive biomarkers capable of predicting poor response to UDCA remains strikingly limited. Since their introduction in 2015 (GLOBE-PBC) and 2016 (UK-PBC), validated prognostic scoring systems with broad clinical credibility remain lacking for primary biliary cholangitis [32,52]. This limitation primarily stems from the low incidence of PBC, which impedes recruitment of large-scale patient cohorts for robust external validation. Furthermore, genetic heterogeneity across ancestries may drive differential treatment responses to UDCA, constraining the predictive utility of these models in diverse populations [53-55]. Furthermore, the field suffers from a notable absence of AI-powered multimodal data integration frameworks for accurate risk stratification. Furthermore, standardized frameworks for evaluating long-term therapeutic efficacy are notably absent. While randomized controlled trials assess novel therapies (e.g., Seladelpar), their typical follow-up periods of < 5 years preclude a robust assessment of sustained disease modification and late-stage outcomes [56].

Bibliometric analyses reveal the critical priorities for advancing PBC research: (1) Multi-omics mechanistic integration: systematic synthesis of single-cell RNA sequencing, spatial transcriptomics, and longitudinal metabolomics data to elucidate spatiotemporal crosstalk between cholangiocytes and hepatic immune niches. (2) Development of prognostic biomarker systems: The irreversible progression of PBC necessitates reliable predictive tools to identify high-risk patients requiring early intervention, thereby preventing end-stage complications, including cirrhosis and liver failure. Future research should focus on identifying novel predictive biomarkers to optimize diagnostic accuracy, therapeutic decision-making, and long-term management in PBC. (3) Future efforts should prioritize international collaboration through standardized electronic health record integration and expansion of multinational registries (e.g., UK-PBC, GLOBAL-PBC). Such infrastructure will: (1) enable validation of clinical guidelines across diverse populations, (2) facilitate pre-registered observational studies with protocol transparency, and (3) establish FAIR (Findable, Accessible, Interoperable, Reusable) data repositories to accelerate open science in PBC research.

Limitations

There are unavoidable limitations. Firstly, while our analysis comprised 2,042 English-language articles from Web of Science-indexed core journals spanning 5 decades, this sample may not fully represent the entire spectrum of PBC immunological research. Secondly, language bias requires critical acknowledgment. While Web of Science-indexed core provides rigorous coverage of high-impact English literature, it excludes databases housing regional journals (e.g., Japan Medical Abstracts Society for Japanese studies, CNKI for Chinese research). This may underrepresent specific geographic perspectives-particularly regarding genetic heterogeneity in Asian populations, traditional medicine approaches, and real-world treatment patterns. Future bibliometric studies should incorporate multilingual databases (e.g., Scopus, Dimensions) to mitigate this gap. Secondly, during the manual screening process, the extensive dataset and broad thematic scope, particularly including articles that addressed autoimmune mechanisms without explicit PBC terminology, inevitably led to the unintentional exclusion of some relevant publications despite rigorous screening protocols. Finally, historical standards may limit methodological rigor in earlier publications, and the nascent stage of recent investigations may preclude robust citation metrics, potentially impacting their bibliometric prominence within our analysis.

Notwithstanding these limitations, our study constitutes the most comprehensive bibliometric analysis of PBC immunology research to date. By systematically mapping five decades of scientific output, establishing a foundational reference point, and delineating critical knowledge gaps and emerging trends to guide future investigations. Looking ahead, advances in machine learning-enhanced bibliometric approaches are likely to yield unprecedented precision in tracking the evolution of PBC research and predicting high-impact directions.

## Conclusions

We performed a comprehensive bibliometric analysis of 2,042 PBC immunology publications (1970-2023) using keyword-based citation network analysis to elucidate evolving research trends and knowledge gaps. We performed a comprehensive quantitative analysis of these articles using VOSviewer and other bibliometric tools. Using bibliometric mapping, we analyzed decades of PBC research publications to reveal the chronological growth patterns, geographical research disparities, and the predominant journal dissemination channels. We also identified the evolving trends in PBC-related topics, investigating the underlying factors driving these changes and the issues they reflect. Advancements in diagnostics and a deeper understanding of PBC have led to more timely diagnoses and improved patient management. Currently, a significant portion of PBC research centers on patient management and emerging treatment options. While considerable progress has been made, critical gaps persist in our understanding of PBC's etiology and pathogenic mechanisms. The convergence of emerging technologies (e.g., spatial transcriptomics, digital pathology) with established research paradigms suggests this field will continue to yield transformative discoveries, positioning PBC as both a model autoimmune disease and a focus of sustained scientific innovation.
